# Assessment of lactoferrin-conjugated solid lipid nanoparticles for efficient targeting to the lung

**DOI:** 10.1007/s40204-015-0037-z

**Published:** 2015-03-27

**Authors:** Satish Shilpi, Vishnoo Dayal Vimal, Vandana Soni

**Affiliations:** 1 Department of Pharmaceutical Sciences, Dr. Hari Singh Gour University, Sagar, 470 003 Madhya Pradesh India; 2 Ravishankar College of Pharmacy, Bhopal, 462 010 Madhya Pradesh India

**Keywords:** Biodistribution, Lung targeting, SLNs, Drug delivery, Lactoferrin

## Abstract

The aim of the present study was to develop a target oriented drug delivery system for the lungs. Lactoferrin (Lf)-coupled solid lipid nanoparticles (SLNs) bearing rifampicin was prepared by a solvent injection method. The prepared nanoparticles were characterized for shape, particle size, polydispersity and percentage drug entrapment. An optimized formulation was then studied for its in vivo performance in animals and to determine its targeting efficiency. It was observed that, upon coupling with Lf, the size of SLNs increased while the percent entrapment efficiency decreases. In in vitro release, determined by a dialysis technique, analysis showed that uncoupled SLNs exhibited higher drug release as compared to coupled SLNs. An in vivo biodistribution study shows 47.7 ±0.4 drug uptakes by the lungs, which was 3.05 times higher in comparison to uncoupled SLNs. These biodistribution studies are further supported by the fluorescence study that revealed enhanced uptake of Lf-coupled SLNs in the lung. From the presented results, it can be concluded that Lf-coupled SLNs enhanced drug uptake in the lung. Moreover, lactoferrin is an efficient molecule that can be used for targeting active agents directly to the lung.

## Introduction

The success of novel drug delivery systems depends on the development of formulations that are capable of improving the therapeutic index of biologically active molecules by increasing their concentration specifically at desired target sites or organs. It is well known that various novel carriers have been used for drug delivery to the lungs for the treatment of tuberculosis, for example, microparticle (Zhou et al. 2005; Zhuang et al. 2012) poly(lactic-co-glycolic acid) (PLGA) nanospheres (Tomoda et al. 2005; Tomoda and Makino 2007), stealth liposomes (Deol and Khuller 1997), functionalized nanoparticles (Sharma et al. 2004), and SLNs (Anisimova et al. 2000; Pandey et al. 2005; Pandey and Khuller 2005). Microparticles offer excellent aerodynamic properties and their large geometric size reduces their uptake by alveolar macrophages, making them a suitable carrier for sustained drug release in the lungs. Similarly, nanocarriers offers significant potential for prolonged drug release in the lungs because they largely escape uptake by lung-surface macrophages and can remain in the pulmonary tissue for a long time. Conjugation of drugs to polymers as polyethylene glycol or stealth liposomes can be particularly beneficial for sustaining the release of drugs. Liposomes can be prepared with lipids endogenous to the lungs and are particularly safe. Their composition can be adjusted to modulate drug release and they can encapsulate both hydrophilic and lipophilic compounds with high drug loading (Loira-Pastoriza et al. 2014).

Among the above, SLNs may be an alternative drug carrier system that offers several advantages conferred by their colloidal dimensions, including: easy incorporation of both lipophilic and hydrophilic drugs; improved biocompatibility; flexible surface functionality; nanoscopic structure; monodispersity; and high encapsulation efficiency (Muller and Keck 2010; Alpiaz et al. 2008; Carneiro et al. 2012; Jaspart et al. 2005; Shuhendler et al. 2012; Wang et al. 2012; Yang et al. 2012).

It is well known that the targeting of the lung offers a challenge due to the mucocilliary clearance. In this context, the ligand-anchored drug delivery system proves its potential in achieving enhanced site-specific drug delivery as well as reduced reticular endothelial system (RES) uptake (Sahu et al. 2014; Baek and Cho 2013; Makino et al. 2004). However, the clinical success of such an approach depends on the selection of appropriate ligands lacking immunogenic potential with the ability to mediate cargo internalization by the target cell (Allen 2002). With this concern in mind, lactoferrin (Lf) may function as a ligand suitable for coupling with SLNs, generating a promising drug delivery system to the lungs.

Lactoferrin is an 80-kDa iron-binding glycoprotein of the transferrin family. Lf is thought to play a role in innate defense and exhibits a diverse range of biological activities, including antimicrobial, antiviral, antioxidant, immunomodulation, modulation of cell growth, and binding and inhibition of several bioactive compounds such as lipopolysaccharide and glycosaminoglycan. Bovine Lf has been found to significantly inhibit colon, esophagus, lung and bladder carcinogenesis in rats when administered orally in the post-initiation stages (Tsuda et al. 2002). Lf receptors are expressed on the apical surface of bronchial epithelial cells and this conception is utilized to achieve targeted drug delivery to the lungs (Elfinger et al. 2007).

To achieve site specificity, Lf was used as a ligand in present project. Chemical cross linking strategies were utilized for the conjugation of Lf with SLNs and the –NH_2_ group present at the surface of stearylamine containing SLNs conjugated with the –COOH groups of Lf. Lf-coupled SLN enhances drug delivery to the lungs because the receptors of this protein are over expressed in the lungs (Schubert and Goymann 2003). Hence, this system may become a promising tool for enhancing drug delivery to treat lung-associated diseases.

Recently, multiple drug chemotherapy has formed the backbone of antituberculotic therapy. Particularly, rifampicin is the first choice drug in the treatment of tuberculosis. But the current treatment of tuberculosis involves prolonged oral administration of large systemic doses of combined antibiotics that are associated with unwanted side effects and poor patient compliance (Ito and Makino 2004; O’Hara and Hickey 2000).

Therefore, the current work was aimed to design and evaluate the efficacy of Lf-anchored rifampicin loaded SLNs for effective management of tuberculosis. In this project Lf was conjugated on the surface of SLN, in which the –NH_2_ group of stearylamine containing SLNs were conjugated with the –COOH groups of Lf. Lf-coupled SLNs enhance lung cancer cell specific (lactoferrin receptor overexpressing cells) targeting and drug delivery. The present study reveals the targeting potential of Lf-coupled SLNs for site-specific delivery of rifampicin to the lungs.

## Materials

The drug (rifampicin) was obtained as a gift sample from Park Pharmaceuticals, Panchkula. Tristearin, soya lecithin, stearylamine, Triton X-100, Tween 80, Sephadex G-50 and 1-ethyl-3-(3-dimethylaminopropyl) carbodiimide hydrochloride (EDC) were purchased from Sigma Chemicals (St Louis, MO, USA). Dialysis membranes (molecular weight cut off 3500) were purchased from Himedia, Mumbai, India. All other reagents and solvents were of analytical grade.

### Preparation of solid lipid nanoparticles (SLNs)

#### Preparation of the uncoupled SLNs

**Fig. 1:**
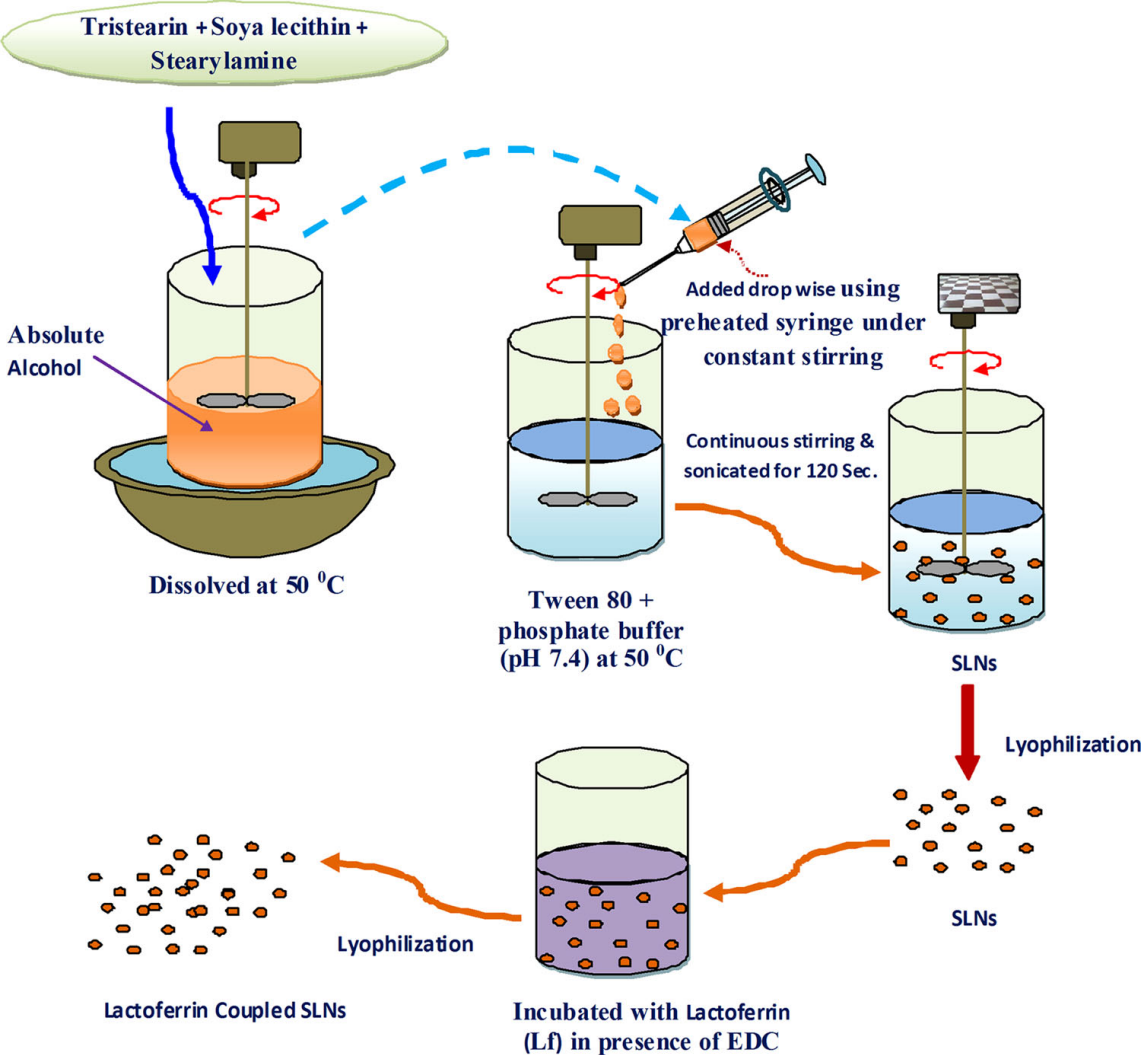
Schematic diagram for SLN preparation

SLNs were prepared according to the solvent injection method previously developed by Schubert and Goymann (2003). In this method, tristearin (100 mg), soya lecithin (100 mg) and stearylamine (10 mg) were dissolved in a minimum quantity of absolute alcohol in different ratios and heated to about 50 °C. Tween 80 (0.5 % v/v) containing phosphate buffer (pH 7.4) solution was heated separately at the same temperature and was used as the aqueous phase. Then the organic phase containing a lipid mixture was added using a preheated syringe to an aqueous solution at the same temperature with continuous stirring for a definite time period. The lipid suspension was then sonicated by probe sonicator of an 10ϕ-amplitude lever at a 20-s pulse rate (power output 2 KW) and a 20-kHz frequency for 120 s, which gives uniformity in the size of the SLNs. Drug-loaded SLNs were prepared by the same procedure in which the drug (10 mg) was dissolved in phosphate-buffered saline (PBS; pH 7.4) to obtain a 10:100 drug:lipid ratio. The complete methodology for preparation of SLNs was shown in Fig. 1.

#### Conjugation of SLNs with lactoferrin (Lf)

Coupling of SLNs with Lf was performed according to the method reported by Gupta et al. (2007) via carbodiimide chemistry (Wissing and Muller 2002), i.e., coupling of the Lf carboxylic group with the stearylamine amine group present on the surface of the previously formed drug-loaded SLNs in the presence of *N*-ethyl-*N*-(dimethylaminopropyl)-carbodiimide (EDC). In preparation of coupled SLNs, the drug-loaded formulations (100 mg) were suspended in a PBS (pH 7.4; 10 ml) containing Lf (10 mg) and EDC (10 mg) and then incubated for 2 h at room temperature. Plain SLNs were removed by passing the formulation through a Sephadex G-50 mini column.

### Characterization of uncoupled and coupled SLNs

#### Particle size, polydispersity index and zeta potential

The average particle size and polydispersity index (PDI) of the SLNs were determined by photon correlation spectroscopy using a Zetasizer DTS, version 4.10 (Malvern Instrument, UK). The formulations were diluted to 1:9 v/v with deionized water. The particles size and PDI were represented by the average diameter of the Gaussian distribution function in the logarithmic axis mode. Particle size, size distribution and zeta potential of the SLN formulation was performed at the National Institute of Pharmaceutical Education and Research (NIPER), Mohali, India.

Surface charge measurement of the SLNs was based on the zeta potential (*ε*) that was calculated according to Helmholtz–Smoluchowsky from their electrophoretic mobility. For measurement of zeta potential, a Zetasizer was used with a field strength of 20 V/cm on a large bore measures cell. Samples were diluted with 0.9 % NaCl adjusted to a conductivity of 50 μS/cm (Nassimi et al. 2010). The results are given in Table 1.

**Table 1:** Various parameters of uncoupled and coupled SLNs bearing rifampicin

S. No.	Formulation	Particle size (nm)	PDI	Zeta potential (mV)	Entrapment efficiency (%)	Coupling efficiency (%)
1	Uncoupled SLNs	235 ± 2	0.076	22 ± 1	73.4 ± 3	Nil
2	Lactoferrin-coupled SLNs	271 ± 2	0.124	23 ± 2	68.4 ± 2	22.7

Mean (*n* = 3) ± SD

### Shape and surface morphology

**Fig. 2:**
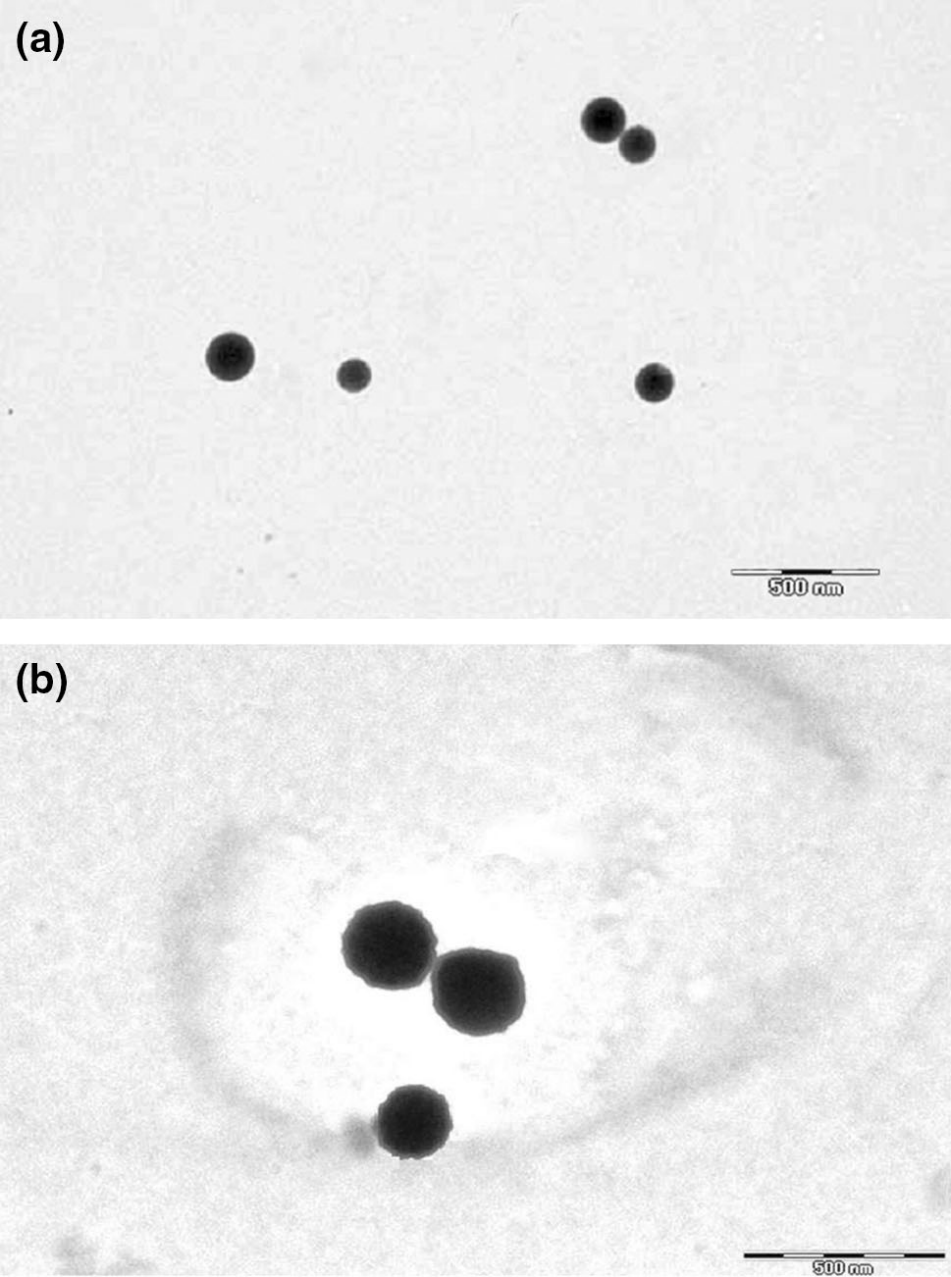
**a** TEM microphotograph of uncoupled SLNs. **b** TEM microphotograph of Lf-coupled SLNs

**Fig. 3:**
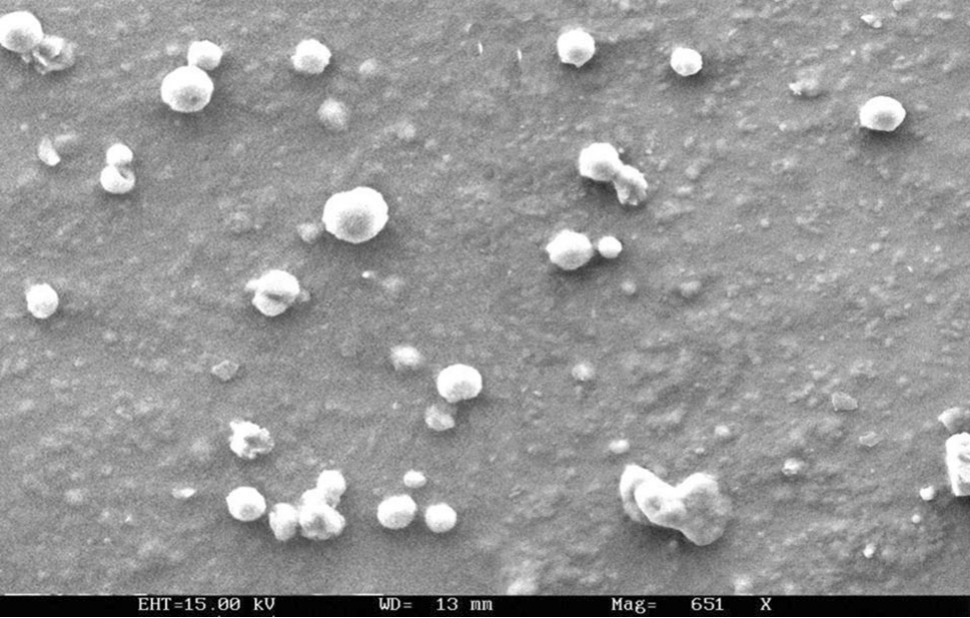
SEM microphotograph of coupled SLNs

In order to examine the SLN surface morphology, the formulations were viewed via scanning electron microscopy (SEM). SEM samples were prepared by lightly sprinkling the lyophilized nanoparticle powder on a double adhesive tape stuck on an aluminum stub. The stubs were then coated with gold to a thickness of about 300 Å using a sputter coater. The photomicrographs were taken with a scanning electron microscope (JEOL JSM-6100). Transmission electron microscopy (TEM; Philips CM12 Electron Microscope, Eindhoven, Netherlands) at an acceleration voltage of 20 kV was used to visualize nanoparticles. Samples were negatively stained with 2 % aqueous solution of phosphotungstic acid. TEM images of uncoupled and coupled Lf are presnted in Fig. 2a, b, respectively. SEM images (Fig. 3) show Lf-coupled SLNs. SEM and TEM were performed at the All India Institute of Medical Sciences (AIIMS), New Delhi.

### Entrapment efficiency

Entrapment efficiency of uncoupled and coupled SLNs was determined using the method described by Gupta et al*. (* 2007) and Fry (1978). The drug not entrapped was removed from the SLNs by passing the formulation through a Sephadex G-50 minicolumn. The weighed amount of Sephadex G-50 was properly mixed with sufficient amount of distilled water in a beaker and kept for 24 h for complete swelling. After complete swelling, Sephadex dispersion was placed in a 1-ml PVC syringe (Dispovan) packed with glass wool and a small piece of Whatman filter paper at the bottom end to provide stability for the Sephadex column at 3,000 rpm. The amount of drug not entrapped in the SLNs was determined by passing the formulation from the Sephadex column, centrifuging at 3,000 rpm, and collecting the elution using the equation from Gupta et al. (2007). After removing the un-entrapped drugs, the SLNs were collected and lysed using 1 % Triton X100; drug entrapment was then analysed spectroscopically.

$$ \% \,\,{\text{Drug}}\,{\text{entrapment}}\, = \,\frac{{{\text{Theoretical}}\,{\text{drug}}\,{\text{content}}\, - {\text{Practical}}\,{\text{drug}}\,{\text{content}}}}{{{\text{Theoretical}}\,{\text{drug}}\,{\text{content}}}} \times \,100\, $$

This solution was then diluted ten times with PBS (pH 7.4) and analyzed with a spectrophotometer at *λ*_max_ of 476 nm. The same procedure was applied for determining drug entrapment of the Lf-coupled SLNs. The percentages of drug entrapment in uncoupled and coupled SLNs are recorded in Table 1.

### In vitro drug release

**Fig. 4:**
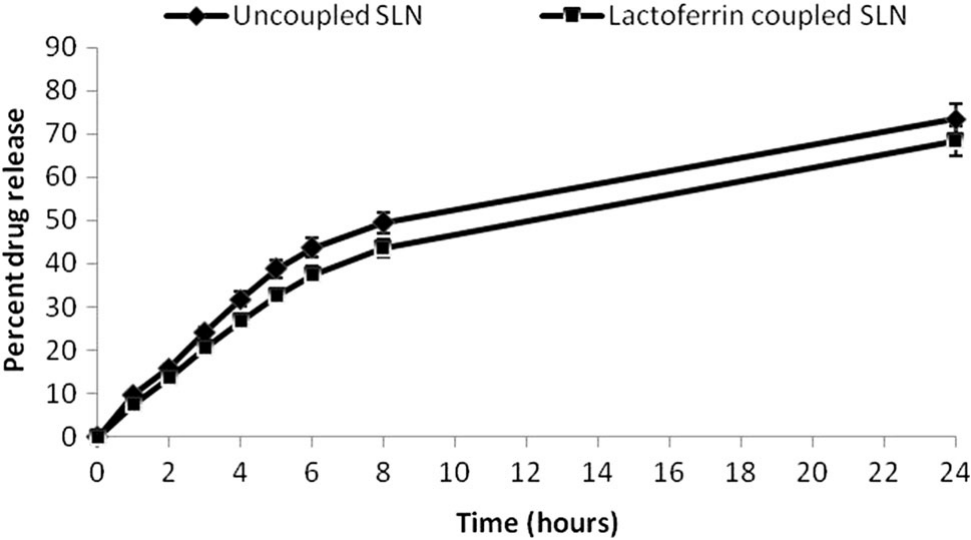
In vitro drug release of uncoupled and coupled SLNs

The drug release of SLNs and Lf-coupled SLNs was performed in PBS (pH 7.4) using the dialysis bag technique. The dialysis bag retains nanoparticles and allows the free drug into the dissolution media with a molecular weight cut off point 3.5 KD. The bags were soaked in double-distilled water for 12 h before use. One ml of pure SLN formulation containing about 100 mg of the drug in about 150 mg of SLN free of any unentrapped drug was taken in a dialysis bag and placed in a beaker containing 50 ml of PBS (pH 7.4) at 37 ± 1 °C throughout the study. The samples were withdrawn after specified time intervals and replaced with the same volume of PBS (pH 7.4). The withdrawn samples were analyzed for drug content by spectrophotometer at 476 nm (Fig. 4).

### Coupling efficiency

The Lf concentration in the coupled SLNs was determined by the Bradford method of protein estimation with minor modifications (Calleja et al. 2004). Briefly, 1 ml of Lf-coupled SLNs containing about 100 mg of SLN formulation was placed in a volumetric flask with 1 ml (10 %) Coomassie blue G dye solution, and the volume was adjusted to 10 ml with distilled water. To determine the Lf concentration, the absorbance at 595 nm was measured and compared with a blank containing the same amount of dye.

### In vivo organ distribution study

Fasted albino rats (average weight 150–200 gm) were divided into three groups each containing 12 animals. Animals of the first group were kept as a control that received an aqueous solution of free drug, while the second and third groups received uncoupled and coupled formulations, respectively. A dose of 1.5 mg/kg body weight was given to the rats intravenously. All studies were carried out according to the guidelines of the Council for the Purpose of Control and Supervision of Experiments on Animals (CPCSEA), Ministry of Social Justice and Empowerment, Government of India and approved by the University Animal Ethical Committee, Sagar (MP), India and performed at the Department of Pharmaceutical Sciences, Dr. H. S. Gour University, Sagar, India.

**Fig. 5:**
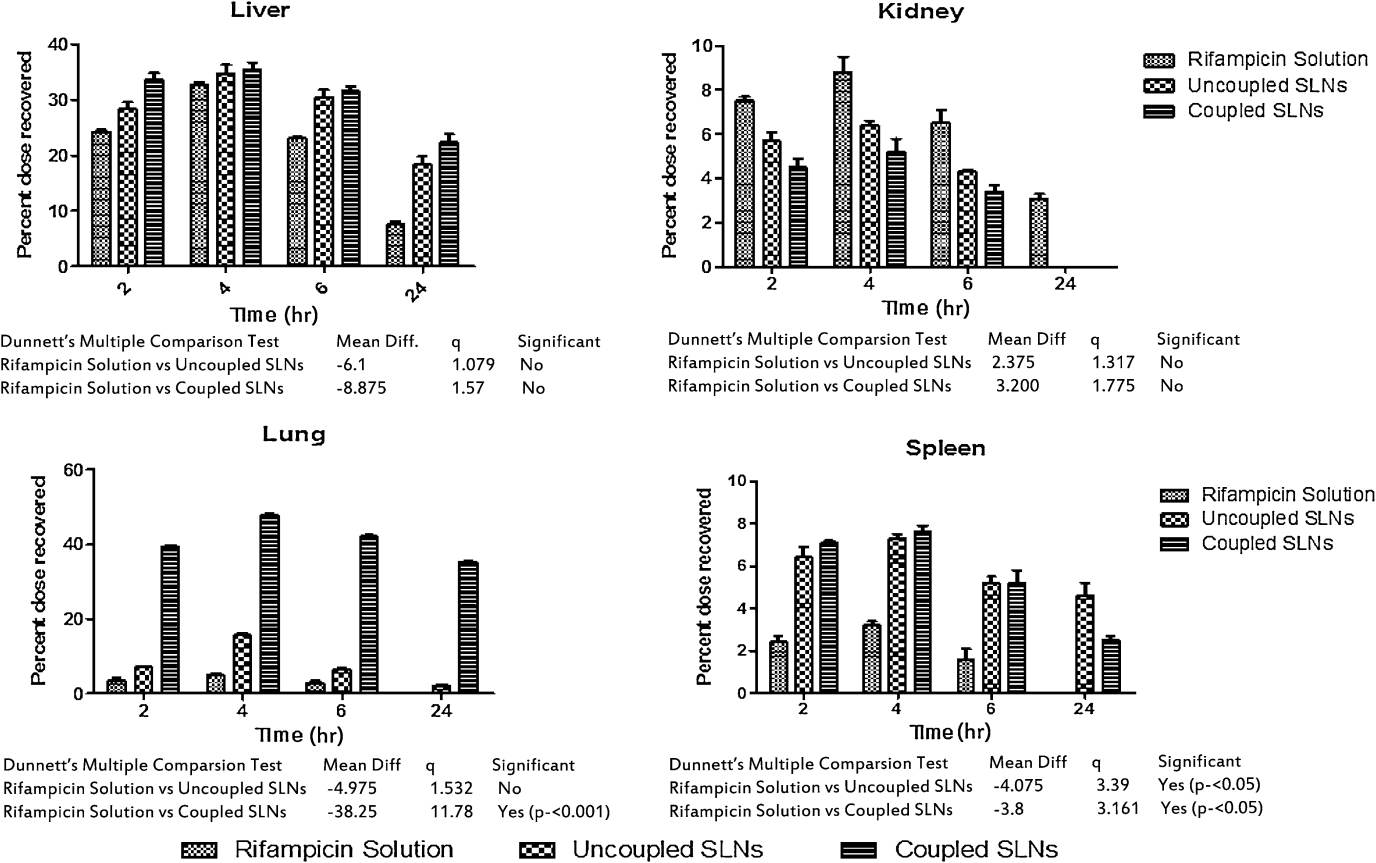
Percent dose recovered in different organs from various formulations, consists of statistically significant values

After administration of the formulations, rats from each group were sacrificed after 2, 4, 6 and 24 h. One gram of each organ was homogenized with 2 ml of PBS (pH 7.4) using a homogenizer. In the case of organs weighing <1 g, the whole organ was used and the amount of drug present in each organ and blood sample was determined by using the high performance liquid chromatography (HPLC) method reported by Calleja et al. (2004) (Fig. 5) (Calleja et al. 2004; Kar 2005). The mobile phase consisted of water (pH 2.27 adjusted with orthophosphoric acid)–acetonitrile (40:60, v/v) at a flow-rate of 1 ml/min. Chromatography was carried out at 25 °C and the elute was monitored at 333 nm on a C_18_ (4.6 × 250 mm, 5 µm) column with a UV detector.

### Estimation of rifampicin in blood plasma

**Fig. 6:**
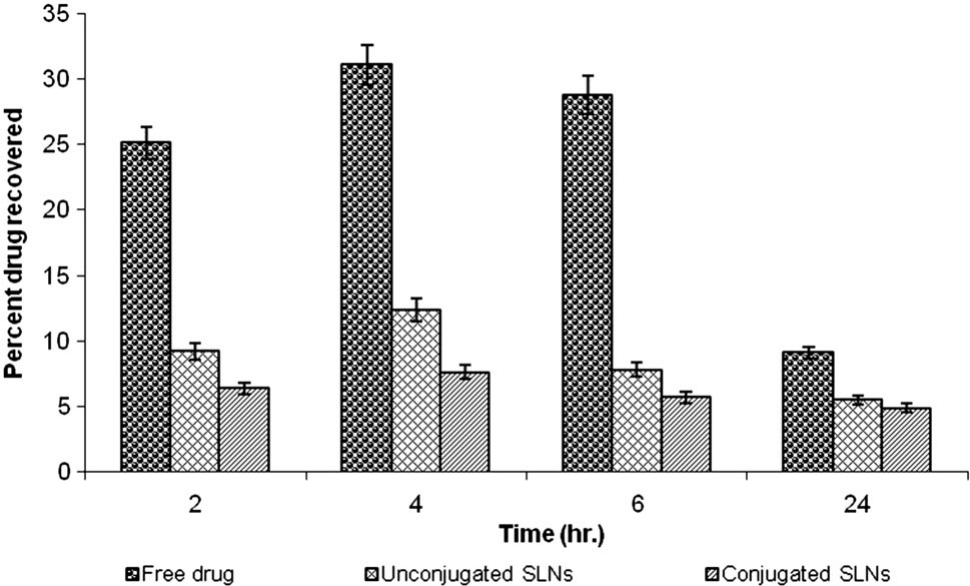
Percent drug recovered in blood plasma from various formulations

Blood was collected through a cardiac puncture in a centrifuge tube containing heparin (anticoagulant) and centrifuged at 2,000 rpm for 15 min. Acetonitrile (1 ml/ml) was added to the in supernatant to precipitate the proteins; the solution was then centrifuged at 2,000 rpm for 15 min and supernatant was collected a second time. The supernatant was filtered through a 0.45-μm membrane filter and analysed as per the procedure used for the organ distribution study. Estimation of rifampicin was done by HPLC as described above and is shown graphically in Fig. 6.

### Fluorescence microscopy

**Fig. 7:**
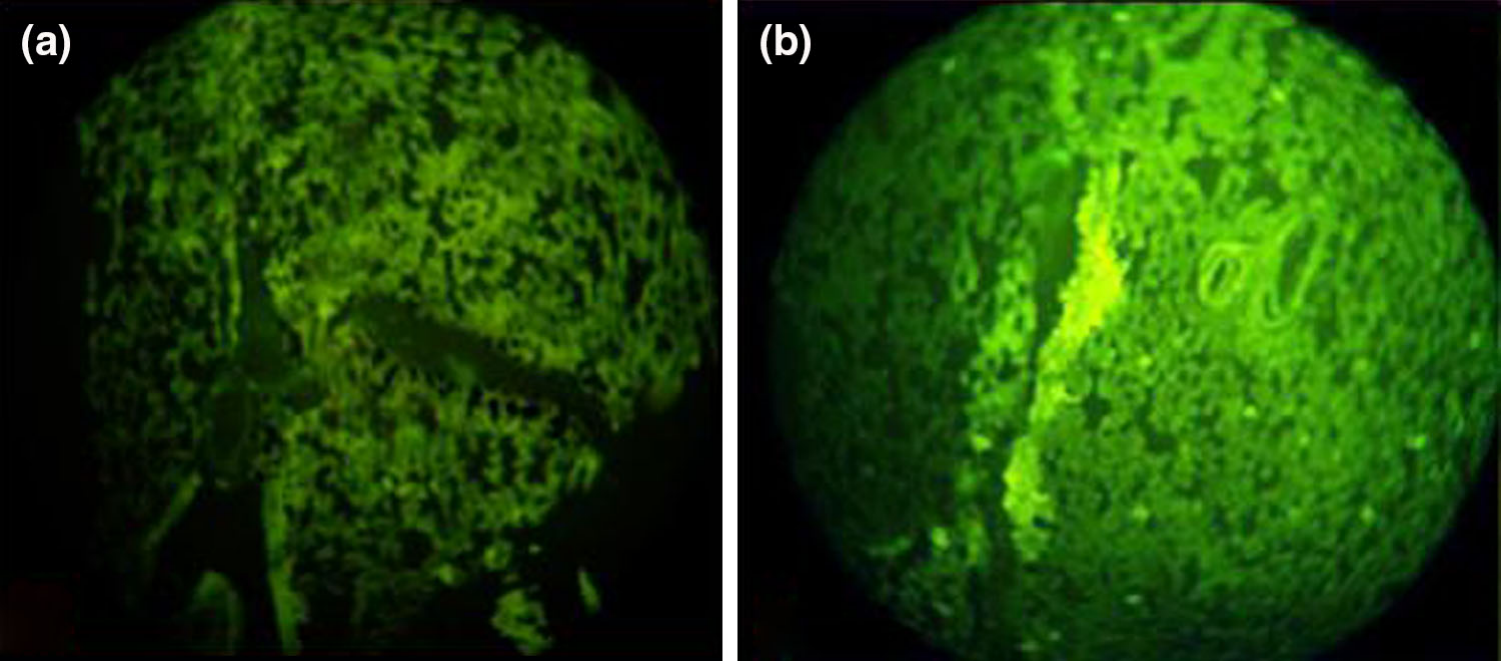
Fluorescence photomicrographs after administration of uncoupled (**a**) and coupled SLNs (**b**)

Fluorescence microscopy was performed in order to confirm the lung uptake of the Lf-coupled SLNs. Rhodamine 6G was used as the fluorescence marker, which was encapsulated into uncoupled and coupled SLNs. The formulations were administered intravenously and sacrificed after 60 min; lungs were then excised and isolated. They were cut into small pieces and washed in Ringer’s solution with subsequent drying using tissue paper. Dried pieces of various organs were fixed in Carnay’s fluid (absolute alcohol:chloroform:glacial acetic acid, 3.5:1:0.5). Then, microtomy was done and ribbons of the sections obtained were fixed on the slides using egg albumin solutions as fixative. The sections were viewed under fluorescence microscope and their photomicrographs are shown in Fig. 7.

### Statistical analysis

Data are expressed as the mean ± standard deviation (SD) and statistical analysis was carried out employing the one-way analysis of variance (ANOVA) test using the PRISM software (Graph Pad). A value of *P* < 0.005 was considered statistically significant (Fig. 5).

### Results and discussion

**Fig. 8:**
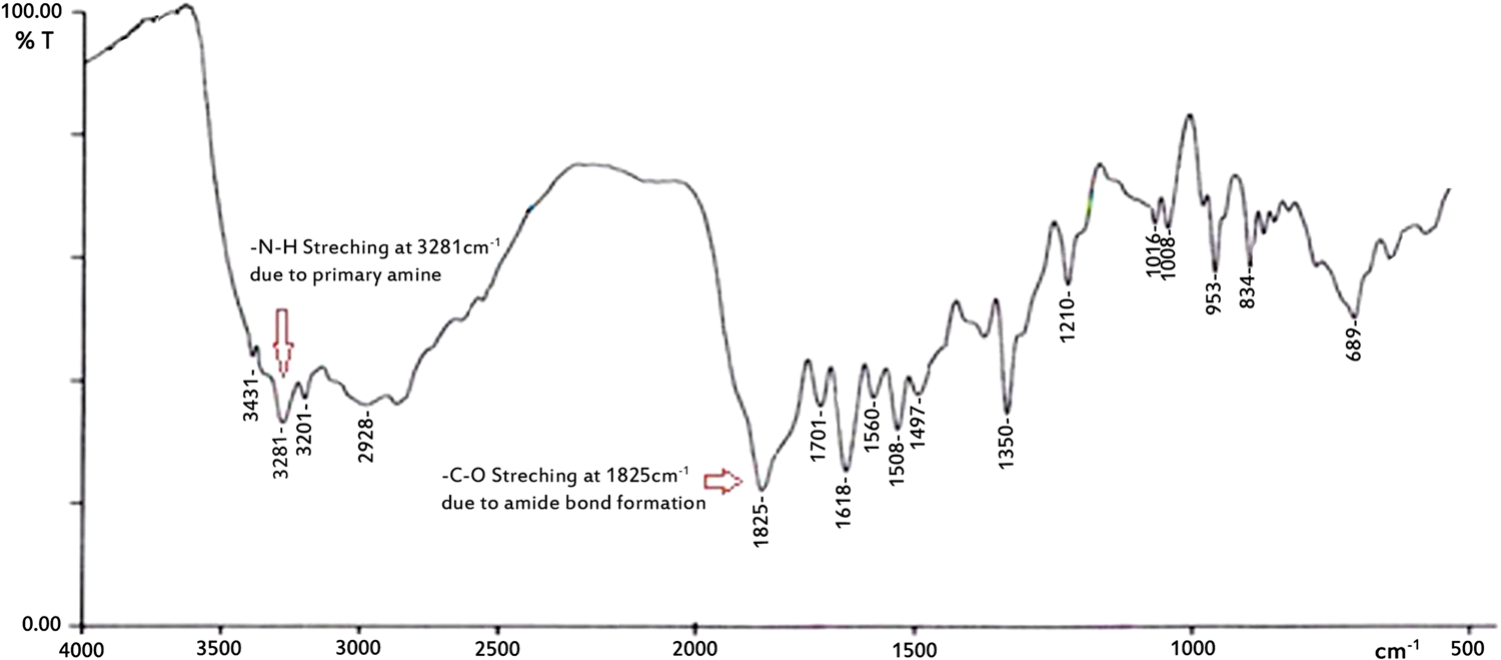
FTIR spectra of lactoferrin-conjugated SLNs

Lf-coupled and uncoupled SLNs were prepared using tristearin, soya lecithin and stearylamine to improve Rifampicin transport to the lungs, and optimized for size, shape and percent entrapment efficiency. Coupling of optimized SLN formulations with Lf was performed using carbodiimide chemistry in which the carboxylate group of Lf conjugated with the amine group of stearylamine present on the surface of previously formed drug-loaded SLNs, with help of EDC as a coupling agent. The coupling of Lf with the SLNs was performed via carbodiimide chemistry; the –CONH-(amide linkage) bond is formed between the –NH_2_ groups present on the surface of the SLNs (due to the presence of stearylamine) and the –COOH group of the Lf. In FTIR spectra, the characteristic peak at 3,288 cm^−1^ represents –N–H stretching of the primary amine, and the peak at 1,625 cm^−1^ represents –C–O stretching. The disappearance of the secondary amine peak in the spectra was additional confirmation of lactoferrin conjugation (Fig. 8).

TEM and SEM studies revealed the spherical shape of coupled and uncoupled SLNs (Figs. 2a, b, 3), whereas the mean diameter of Lf-coupled and uncoupled SLNs was found to be 235 ± 2 and 271 ± 2 nm, respectively (Table 1). The size of the coupled formulation was found to be higher when compared to the uncoupled formulation, which could be due to the coupling of Lf on the surface of the SLN. The PDI was found to be <0.2 for both formulations, indicating a narrow size distribution of particles and, consequently, a uniform distribution. The zeta potential of uncoupled and coupled SLNs was found to be 22 ± 1 and 23 ± 1 mV, respectively (Table 1). The zeta potential of both formulations was found to be similar, which indicates that Lf does not markedly affect the cationic surface charges of SLNs. The drug entrapment efficiency of Lf–SLNs was found to be less as compared to uncoupled SLNs. For uncoupled SLNs, the entrapment efficiency was found to be 73.4 ± 3 % and for Lf–SLNs, it was 68.4 ± 2 %. This may be due to leaching of any residual drug during Lf coupling in the drug-loaded SLN formulation. The coupling efficiency of Lf-SLNs was found to be 22.7 %.

In vitro drug release from coupled and uncoupled SLNs was studied using the dialysis technique. The percent drug release from the uncoupled and coupled SLNs in PBS (pH 7.4) at different time intervals was recorded and, for the former, it was found to be 73.4 ± 3 %, whereas in the case of the last, it was 68.4 ± 2 % in 24 h. The decrease in drug release profile for the coupled formulation may be due to structural integrity conferred by Lf coupling that might lead to a double barrier effect for drug diffusion.

Blood plasma level studies of formulations were conducted to determine their release and performance in vivo. Blood plasma was used to determine the concentration of the drug in blood samples at various time intervals. Intravenous administration of free rifampicin resulted in a very high drug concentration in the plasma; it was found to be 31.04 ± 0.3 % after 4 h, which rapidly decreased to 9.1 ± 0.7 % after 24 h. This may be due to rapid excretion of the drug from the kidney and simultaneous distribution of the drug in various organs. However, the concentration of the drug in plasma after administration of different types of SLNs (uncoupled and coupled) was considerably low. For the uncoupled formulation, the maximum percent dose recovered in plasma was found to be 12.4 ± 0.2 % after 4 h, which decreased to 5.5 ± 0.4 % after 24 h. For the coupled formulation, the maximum percent dose recovered in plasma was found to be 7.6 ± 0.7 % after 4 h, which decreased to 4.9 ± 0.2 % after 24 h (Fig. 5). The significant reduction in drug concentration in blood plasma may be due to the fact that most of the drug present in blood was entrapped in SLNs, leading to a reduced rifampicin toxicity; also, the method by which the drug was extracted from the plasma did not extract the drug from the SLNs.

The lung uptake of both coupled and uncoupled SLNs was assessed via a biodistribution study after intravenous administration of a solution of free drug in coupled and uncoupled formulations. The results of the biodistribution studies exhibit a maximum accumulation of the drug in the liver, kidney and spleen after intravenous administration of a plain drug. The concentrations of drug accumulated in different organs following intravenous administration of a plain drug after 4 h were found to be 32.7 ± 0.5 % in the liver, 8.8 ± 0.7 % in the kidney, 4.9 ± 0.4 % in the lung and 3.2 ± 0.2 % in the spleen, each of which were declined. After 24 h, the amount of drug recovered was found to be 10.6 ± 0.5 % in the liver and 3.1 ± 0.2 % in the kidney, respectively, while the quantity of drug was not detectable in the lung and spleen (Table 2). In the case of the uncoupled SLN formulation, the percentage of drug recovered by the liver and spleen was found to be 34.8 ± 1.6 and 7.3 ± 0.2 % after 4 h and 18.4 ± 1.5 % and 4.6 ± 0.6 after 24 h, respectively. Whereas, in the case of the coupled formulation, the percent drug recovered by the liver and spleen was found to be 35.5 ± 1.3 and 7.6 ± 0.3 after 4 h and 22.4 ± 1.5, and 2.5 ± 0.2 after 24 h, respectively. The decrease in percent drug recovery may be due to less RES uptake of the drug from the liver and spleen in the case of coupled formulation. The percent drug recovered by the lung was found to be 15.6 ± 0.6 after 4 h and 2.1 ± 0.5 after 24 h in the case of the uncoupled formulation, while with the Lf-coupled formulation, the percent drug recovered was found to be 47.7 ± 0.4 and 35.3 ± 0.2 after 4 and 24 h, respectively. The results show that there was approximately a threefold increase in lung uptake of coupled SLNs as compared to uncoupled formulations. The increased drug uptake from coupled SLNs by the lung, compared to the free drug solution and uncoupled SLN formulation, may be due to Lf being recognized by the receptor present on the cell membrane of alveolar epithelial cells of lung tissue. This observation could be due to a greater abundance of Lf receptors on lung tissue.

**Table 2:** Organ drug distribution from various formulations

	Time (h)	Percent dose recovered			
		Liver	Kidney	Lung	Spleen
Rifampicin drug solution	2	24.3 ± 0.3	7.5 ± 0.2	3.6 ± 0.7	2.4 ± 0.3
	4	32.7 ± 0.5	8.8 ± 0.7	4.9 ± 0.4	3.2 ± 0.2
	6	23.1 ± 0.2	6.5 ± 0.6	2.9 ± 0.4	1.6 ± 0.5
	24	7.6 ± 0.5	3.1 ± 0.2	Nil	Nil
Uncoupled SLNs	2	28.4 ± 1.2	5.7 ± 0.4	7.1 ± 0.3	6.4 ± 0.5
	4	34.8 ± 1.6	6.4 ± 0.2	15.6 ± 0.6	7.3 ± 0.2
	6	30.5 ± 1.4	4.3 ± 0.1	6.5 ± 0.2	5.2 ± 0.3
	24	18.4 ± 1.5	Nil	2.1 ± 0.5	4.6 ± 0.6
Coupled SLNs	2	33.7 ± 1.2	4.5 ± 0.4	39.3 ± 0.5	7.1 ± 0.1
	4	35.5 ± 1.3	5.2 ± 0.6	47.7 ± 0.4	7.6 ± 0.3
	6	31.6 ± 0.8	3.4 ± 0.3	42.1 ± 0.4	5.2 ± 0.6
	24	22.4 ± 1.5	Nil	35.3 ± 0.2	2.5 ± 0.2

SD ±  mean (*n* = 3)

Dunnett’s test was applied for comparing Lf-coupled and uncoupled SLN formulations with a rifampicin solution as a control; it was found that there was no significance for the liver and kidney, but, for the lung, a value of *P* < 0.001 was considered statistically significant when Lf-coupled SLNs were compared with uncoupled SLNs (Fig. 5).

Fluorescence photomicrographs (Fig. 7) showed the qualitative uptake and the localization pattern of SLNs in the lung. The Lf-coupled SLNs were loaded with rhodamine 6G and were administered intravenously to albino rats; the lung was then isolated after 60 min from a sacrificed animal and photomicrographs were taken. The alveolar macrophages were distinctly filled with the formulation, while other cells, blood capillaries and thick junction places depict diffuse fluorescence as shown in the photomicrograph. The photomicrographs (Fig. 7b) clearly show access of the Lf-coupled SLNs into the lung.

## Conclusion

The aim of the study was to design SLNs loaded with the anti-tuberculosis drug rifampicin. One factor determing the success of a drug delivery system is a higher concentration of the drug at the site of action with decreased side effects to the non-target tissues. Prepared SLNs were coupled with Lf to achieve target-specific delivery of rifampicin to the lungs. In vitro drug release and biodistribution studies of rifampicin-loaded coupled and uncoupled Lf showed that the Lf-coupled formulation sustains the drug release as well as delivers the drug at a higher concentration to the target. Based on these results, it can be concluded that the formulations developed in this work may be considered as effective drug delivery systems for the treatment of tuberculosis and other lung-associated diseases.This work, however, requires further experimental study.
